# Effect of *GH* p.L127V Polymorphism and Feeding Systems on Milk Production Traits and Fatty Acid Composition in Modicana Cows

**DOI:** 10.3390/ani10091651

**Published:** 2020-09-14

**Authors:** Salvatore Bordonaro, Serena Tumino, Donata Marletta, Anna De Angelis, Fortunato Di Paola, Marcella Avondo, Bernardo Valenti

**Affiliations:** 1 Department of Agriculture, Food and Environment, University of Catania, Via Valdisavoia 5, 95123 Catania, Italy; s.bordonaro@unict.it (S.B.); serena.tumino@unict.it (S.T.); d.marletta@unict.it (D.M.); deangeli@unict.it (A.D.A.); fortunato.dipaola@gmail.com (F.D.P.); 2 Department of agricultural, food and environmental Science, University of Perugia, Borgo XX Giugno 74, 06121 Perugia, Italy; bernardo.valenti@unipg.it

**Keywords:** *GH* gene, modicana cows, pasture feeding, milk quality, milk fatty acids

## Abstract

**Simple Summary:**

Growth hormone (GH) participates in the regulation of lactation and lipid metabolism. Milk traits and fatty acid composition were investigated in Modicana cows, in relation to the genetic polymorphism at *GH* locus p.L127V and its interaction with the feeding system. It has been compared 8 hours of grazing without concentrate (EX), versus 2 h of grazing with concentrate (SI). The frequencies of LL, LV and VV genotypes were, respectively, 0.64, 0.34, 0.02. The GH polymorphism and its interaction with feeding system did not influence milk yield and gross composition. LL cows showed higher levels of total unsaturated and monounsaturated fatty acids, thus suggesting a potential role of the GH polymorphism on improving the healthy quality of milk. A higher level of 18:1 in the LL cows was evident only in the extensive system. The lower energy input in the extensive system, compared to the semi-intensive system, could justify this result.

**Abstract:**

Growth hormone participates in the regulation of lactation and lipid metabolism. A trial study was conducted to evaluate the effects of genetic polymorphism at GH p.L127V and its interaction with feeding system (extensive, EX; semi-intensive, SI) on milk traits and fatty acids composition in Modicana cows. In the semi-intensive farm (SI) diet consisted of hay, concentrate and 2 h of grazing. In the extensive farm (EX) feeding consisted in 8 h of grazing and hay. The frequencies of LL, LV and VV genotypes were, respectively: 0.64, 0.34, 0.02. GH polymorphism and its interaction with feeding system did not influence milk yield and composition. Cows carrying LL genotype produced milk with lower 6:0 and 8:0 and higher 16:1 *c*9 and 18:1 *c*9, total UFA and total MUFA. Feeding significantly affected fatty acids: in EX cows lower SFA and higher PUFA and UFA were found, compared to SI cows. The lower, more favorable atherogenic index of milk from EX system was coherent with the improved healthy characteristics of milk from animals fed almost exclusively on pasture. A significant interaction genotype x feeding system was evident for 18:1, higher in the LL cows only in the EX system, but not in the SI system.

## 1. Introduction

Growth hormone (GH) is a member of the somatotropin/prolactin family of hormones secreted in acidophilic cells of the anterior pituitary gland of mammals. It plays an important role in growth, lactation, milk production, reproduction processes, protein, lipid and carbohydrate metabolism [1,2,3,4]. As a result of these physiological effects, GH is considered a candidate gene for marker-assisted selection in livestock [5,6,7]. The bovine growth hormone (*GH*) gene (Gen Bank Acc. Num. M57764.1) is located in BTA19; it includes five exons that encode a polypeptide of 217 amino acids [8,9]. Among the different polymorphisms characterized at the *GH* locus, the missense single nucleotide polymorphism (SNP) C > G in exon 5, that changes leucine to valine in position 127, is of particular interest [10]. Many studies have focused on this polymorphic site, named GH p.L127V, in order to evaluate the allele frequency distribution in dairy, meat and dual-purpose cattle breeds. Schlee et al. [11] reported that the leucine homozygous genotype (p.127LL) was associated with a higher GH concentration in the bloodstream. The research has investigated the effects of the polymorphism GH p.L127V on production traits, such as milk production, growth or carcass traits in cattle. The influence of GH p.L127V polymorphism on milk production traits is not conclusive, and seems to be rather breed-specific [12,13]. Furthermore, the p.127V variant is present at low frequency in many breeds; therefore, the association analysis is often limited by the lack of homozygous p.127VV animals, which disables a direct comparison of both homozygous variants.

Growth hormone is involved in lipid metabolism [14]. Due to this effect, a direct or indirect role of GH on the acidic composition of milk could be hypothesized.

The role of pasture feeding on milk traits and fatty acid composition is well known. Milk fat from ruminants fed with pasture is generally characterized by higher levels of polyunsaturated fatty acids, isomers of conjugated linoleic acid (CLA) and omega-3 fatty acids, compared to milk from stall-fed ruminants [15].

In Italy, despite of the wide diffusion of cosmopolite specialized breeds, several local cattle breeds are still farmed in marginal areas [16]. Most of them are traditionally reared in extensive and semi-intensive systems. In Sicily, the Modicana breed is reared for milk production, that is mainly addressed to the production of “Ragusano”, a protected designation of origin (PDO) cheese, ageing from 4 to 12 months. During the grazing season, especially in the area of origin, the Hyblaean plateau, feeding system of Modicana cows is essentially based on pasture with no, or limited supplementation of concentrate; however, semi-intensive farming is also present where the time spent on pasture is lower and level of concentrate supplementation is higher than in the extensive system. Despite it is important role in the local economy, the Modicana cattle breed has been scarcely investigated for its genetic polymorphism, except for milk protein genes [17], the *MC1R* gene [18] and, more recently, for some genes involved in lipid metabolism [19].

The aim of this research was to study the effects of the genetic polymorphism GH p.L127V and its interaction with the feeding system on milk traits and fatty acids composition in Modicana cows. In particular, it has been studied if a different level of pasture in the diet could interfere on the effect of GH polymorphism on milk yield and its nutritional quality and acidic profile.

## 2. Material and Methods

The experiment lasted 7 months, from October to April in two Modicana dairy cow farms located in the province of Ragusa (Southern of Italy) and characterized by two different feeding systems: a semi-intensive system, SI (36°56′47′′ N and 14°41′50′′ E; 639 m above sea level) and an extensive system, EX (36°52′53′′ N and 14° 33′51′′ E; 308 m above sea level), In the semi-intensive farm (SI) the diet consisted of stall feeding (hay and concentrate) and 2 h of daily grazing on natural pasture. In the extensive farm (EX) feeding consisted primarily of natural pasture (8 h per day) and hay.

### 2.1. Genetic Characterization

The DNA of 188 cows (specifically 118 from SI and 70 from EX) was obtained from milk samples by using the Milk DNA Preservation and Isolation Kit (Norgen Biotek), following the manufacturer’s instructions. The concentration of extracted DNA was measured by using the NanoDrop 1000 Spectrophotometer and brought to 30–40 ng/μL.

Single Nucleotide Polymorphism C > G at the V exon of the *GH* locus (Acc. Num. M57764.1) has been analyzed by PCR-RFLP according to Komisarek et al. [20]. The polymorphism, which causes an amino acid substitution in the protein sequence, is identified as p.L127V. Amplifications were performed in a 30 μL reaction volume using a GenAmp PCR System 9700 (Applied Biosystems, Foster City, CA, USA) thermal cycler. Amplified fragments 404 bp long were digested for 2 h at 37 °C with 5 units of *AluI* (New England BioLabs Inc., Ipswich, MA, USA). Restriction patterns were visualized and recorded after electrophoretic separation in 3% GelRed Nucleic Acid Gel Stain (Biotium, Inc., Fremont, CA, USA) agarose gel (Bio-Rad Laboratories, Inc., Segrate, Italy) using the ChemiDoc^TM^ System with Image Lab^TM^ Software (Bio-Rad Laboratories, Inc., Segrate, Italy).

The polymorphic region of *GH* exon V was sequenced in six samples (2 for each genotype p.127LL, p.127LV, p.127VV) in order to check potential new SNPs. The fragments of 404 bp were sequenced on both strands on an ABI PRISM 3130 Genetic Analyser, equipped with Sequencing Analysis software (Applied Biosystems, Foster City, CA, USA) and aligned with the reference sequence (GenBank Acc. Num. M57764.1) by using the software CLUSTALX 1.8 [21].

### 2.2. Animals and Experimental Design

According to the 188 observed genotypes at the *GH* locus, two experimental groups were formed. A total of 66 cows, 36 from SI and 30 from EX, at their third or fourth lactation, homogeneous for days of lactation (88.2 ± 22.1 and 95.7 ± 31.2 days for SI and EX, respectively) and milk yield (11.0 ± 3.0 and 12.2 ± 6.3 kg/d for SI and EX, respectively) were selected. Within each farm, the *GH* genotypes were distributed as follows:

- SI: LL, 21 cows; LV, 13 cows; VV, 2 cows;
- EX: LL, 19 cows; LV, 10 cows, VV, 1 cow.

In the two farms, the cows were fed as follows:

SI cows were fed with 5 kg of vetch and oats hay, 5 kg of a commercial concentrate and grazed 2 h per day on natural pasture;

EX cows were fed with 5 kg of vetch and oats hay and grazed 8 h per day on natural pasture.

### 2.3. Data Collection and Chemical Analysis

Every month individual milk productions were recorded and the relative milk samples, consisting of proportional volumes from the morning and evening milk, were collected. Fat, protein and lactose were analyzed using an automated Fourier transform infrared absorption spectrophotometric analyzer (Combi-foss 6000, Foss Electric, Hillerød, Denmark). Milk fat was extracted and converted to fatty acid methyl esters (FAME) [22]. Nonadecanoic acid was used as an internal standard (Sigma-Aldrich, St. Louis, MO, USA). FAME were analysed on a Trace Thermo Finnigan GC equipped with a flame ionization detector and a 100 m × 0·25 mm i.d. fused-silica capillary column (SP-2560, Supelco, Inc., Bellefonte PA, USA) according to Valenti et al., [19]. Fatty acids were expressed as g/100 g of total fatty acids.

### 2.4. Statistical Analysis

Genotypic and allelic frequencies were calculated, and the Chi-squared test was used to test whether the population deviated from Hardy–Weinberg equilibrium. Individual data for milk yield and composition (fat, protein, lactose, fatty acids) were analyzed using the GLM procedure for repeated measures of Statistical Package for Social Science (SPSS for Windows, SPSS Inc., Chicago IL, USA). The analysis included main effects of *GH* genotype, feeding system, period and the interaction genotype × feeding system. Pre-experimental data of daily milk production were used as a covariate for milk yield and gross composition. When the covariance was not significant (*p* > 0.05) it was removed from the statistical model. When interaction was significant, differences between means were assessed using the Tukey’s adjustment for multiple comparisons. Significance was declared when *p* < 0.05.

## 3. Results

In the whole sample of 188 Modicana cows, three *GH* genotypes were found; the distribution of individual genotypes, with genotype and allele frequency in the two farms are reported in Table 1.

The genotype p.127LL was the most frequent in Modicana cows. The genotypes resulted well distributed in the two farms (SI and EX) and no significant deviation from the Hardy–Weinberg equilibrium was observed (Table 1). No new SNPs were found by sequencing the analyzed exonic region of the *GH* gene.

Table 2 reports the main effects of *GH* genotype and feeding system on milk yield and gross composition. Since, in our case, the VV Modicana cows were only three, this genotype was excluded from the statistical analysis. In this sample of Modicana cows milk yield and gross composition were not influenced by GH p.L127V polymorphism.

The feeding system affected milk yield and protein content, that were higher in the SI reared cows, and lactose, that was higher in the EX reared cows. No significant interaction was found between *GH* genotype and feeding system.

Fatty acid composition of milk is reported in Table 3. Most of the fatty acids were not affected by *GH* variants; only 6:0 and 8:0 were significantly lower, whereas 16:1 *c9* and 18:1 *c9*, UFA and MUFA were significantly higher in the LL cows. Atherogenic index (AI) was not influenced by the *GH* genotype; however, there is a non-significant trend (*p* = 0.061) toward a lower value in the LL cows. As expected, the feeding system significantly affected the fatty acid profile: short chain fatty acids (4:0, 6:0 and 8:0), 17:0, 18:0, 18:1 *t11*, 18:3, 18:2 *c*9 *t*11, total PUFA, total UFA and UFA/SFA ratio were higher in cows reared in the extensive system, whereas medium chain fatty acids (12:0, 14:0, 16:0), branched chain fatty acids (15:0 *iso*, 17:0 *iso*, 17 *anteiso*), monounsaturated fatty acids (12:1, 14:1, 16:1) and total SFA were higher in the semi-intensive system. The atherogenic index was significantly higher in the SI system. Significant interaction between genotype and feeding system was evident only for 18:1*cis* which showed significant lower values in LV cows, compared to LL cows, only in the EX system (21.4 vs. 18.4, *p* < 0.05), but not in the SI system (20.3 vs. 20.1, *p* > 0.05).

## 4. Discussions

### 4.1. GH p.L127V Polymorphism in Modicana Cows

In Modicana breed *GH* gene, a potential candidate gene for production traits, has been investigated for the first time. The p.127LL genotype was the most frequent in our sample, whereas p.127V allele was found at low frequency (0.20). This evidence confirmed the previous studies that reported GH p.127V variant as the minor allele in a wide range of *Bos genus* breeds.

In *Bos indicus*, GH p.L127V has been reported to be monomorphic for L allele in 324 Braham steers [24], in diverse populations of Nelore and in small samples of Gyr and Guzerá, two zebuine breeds from Brazil [25,26]. The very low frequency of GH p.127V allele (≤0.15) was found in small samples of Santa Gertrudis and Canchim, two *Bos indicus* × *Bos taurus* crosses reared in Brazil [25] and in some taurine breeds: Brown Swiss [12], Holsteins [5,12,27,28] Guernsey [12], Bavarian and Tyrolean Brown [11], and Italian Podolian [29].

In the present study, the Modicana GH p.L127V polymorphism was similar to that described earlier in German Black and White [11], Ayrshire [12], Jersey [27], and in two rustic Turkish breeds [30]. Instead, considerably higher frequencies of the V allele (≥0.28) were observed in Charolais [25] Piemontese [31], Limousine [32]. According to Shariflou et al. [33] GH p.127L allele was the most frequent in almost all the reported breeds but with averagely lower values in meat breeds. This has led the authors to hypothesize that the superiority in milk production of cows carrying L allele has induced a major selection towards the subjects carrying the L allele in milk breeds, but not in meat breeds. As confirmed by Čítek et al. [34], the spread of both alleles in the German Holstein population was affected indirectly by selection for milk production in the past, resulting in a higher occurrence of L allele.

### 4.2. GH Polymorphism Effects on Milk Yield, Composition and Fatty Acids Content

Lucy et al. [12] reported that GH release in German Black-White cows carrying L genotype was higher than in cows carrying V genotype. Coherently, Sorensen et al. [13] found that LL Danish Jersey calves released 57% more GH than LV calves, which, in turn, released 53% more GH than VV calves. However, this effect seems to be breed-specific since they did not find similar differences in GH release between these genotypes in Danish Red and Holstein breeds.

Taking into account the positive effect of circulating GH on milk yield [35], a production increase would be expected in LL cows. Dario et al. [36] in Jersey and Akyuz et al. [28] in Holstein cows found higher milk production in LL genotype. Coherently, Lucy et al. [12] and Heidari et al. [37] found a genetic advantage for milk production in LL Holstein cows. Dybus [38] found higher milk yield in LL cows only during the first lactation. Shariflou et al. [33] found that LL and LV Australian Holstein-Friesian cows produced more milk than VV cows. Contrasting with the above results, Kovacs et al. [39] found that heterozygous cows (LV) had significantly higher 305 days of lactation milk yield, compared to LL cows. In Modicana cows the GH p.L127V polymorphism did not determine significant variations in milk yield. These results are in line with other authors [40,41].

Conflicting results are also reported on the effects of GH polymorphism on milk gross composition: in our study, no significant effect was evident. Dario et al. [36] found higher milk fat and protein content in LV genotype. Dybus et al. [38] and Kovacs et al. [39] report higher milk fat and protein in LL cows. Yardibi et al., [42] reported that LL genotype had significantly higher milk fat level compared to the other genotypes.

To our knowledge, no studies report the effects of the *GH Alul* polymorphism on milk fatty acid profile. The GH p.L127V polymorphism showed no effects on the fatty acid composition of meat in Korean cattle [43]. To better understand the effects of a greater release of somatotropin on the acid composition of milk fat, it is possible to refer to studies on the role of exogenous use of bovine somatotropin (bST) on this aspect. Taking into account the fact that the LL genotype would appear to be associated with a higher somatotropic hormone release [13], one could hypothesize an analogy of response in the milk fatty acid composition between subjects carrying the LL genotype and subjects treated with exogenous bST.

Linch et al. [44] did not find any difference in cows fatty acid composition as a consequence of treatment with bovine somatotropin. As reported by Lough et al. [45], the treatment of lactating dairy cows with somatotropin did not show any influence on the mechanisms of fatty acid synthesis or mammary lipid metabolism. Marty and Block [46] found a low effect of bST treatment on the milk fatty acid profile, whereas Apps et al. [47] found that cows given bST during late lactation, when in positive energy balance, produced milk with lower levels of C4 and long chain fatty acids and higher levels of medium-chain fatty acids, compared to untreated cows.

Eppard et al. [48] administering subcutaneous injections of 0 IU/d, 5 IU/d, 10 IU/d, 25 IU/d, 50 IU/d, and 100 IU/d of growth hormone report that the secretion of short and medium chain fatty acids in milk was increased but the increase has stabilized between 50 and 100 IU/d, whereas the secretion of long-chain fatty acids progressively increased until the highest level of injection.

In our conditions, Modicana cows carrying the LL genotype produced milk with lower levels of 6:0 and 8:0, but no differences were found for medium and saturated long chain fatty acids. On the contrary, 16:1 *c*9 and 18:1 *c*9, total UFA, and total MUFA were higher in LL cows. These results seem to be in line with the possible negative energy balance induced by a hypothetical increase of GH release in the LL cows. In fact, in general, it has been widely demonstrated that a higher level of GH (for example during a bST treatment) induces a negative energy balance status, due, among other causes, to a lower insulin sensitivity. This metabolic status induces an increase of body fat reserves mobilization and, as a consequence, an increase of monounsaturated fatty acids, especially 16:1 *c*9 and 18:1 *c*9 [49,50]. Indeed, as reported by Palmquist et al. [51], long-chain FA deriving from plasma are incorporated into milk fat, so they inhibit the de novo synthesis of short-chain FA by the mammary gland. As a consequence, one would expect a reduction of the short chain fatty acids, as occurred in our experimental conditions for 6:0 and 8:0. A similar trend of short chain fatty acids as effect of the energy balance is reported by Nogalski et al. [49].

### 4.3. Feeding Effects on Milk Yield and Composition and Fatty Acid Profile

The significantly higher levels of milk yield and protein percentage found in cows fed with hay, concentrate and few hours of pasture are the probable consequence of the higher energy and protein input, compared to cows fed mainly with pasture. Surprisingly no differences were found between feeding systems in the fat percentage. In fact, one would have expected a reduction of milk fat content in the animals that received feeds, such as concentrates, poorer in structural carbohydrates. The significant differences between the two feeding systems for most fatty acids are associated with the different proportion of pasture in the two diets. In fact, it is well known that the role of pasture feeding on milk fat composition: the higher levels of 14:1, 16:0 and total SFA and the lower levels of 18:0, 18:1 *t*11, 18:2 *c*9 *t*11, 18:3, branched chain fatty acids (15:0 *iso*, 17:0 *iso* and 17:0 *anteiso*) total UFA and total PUFA in cows fed in the semi intensive system are in line with results reported for stall fed cows, compared to pasture fed cows, by Villeneuve et al. [52]. The higher UFA/SFA ratio and the lower, more favorable atherogenic index of milk from the EX system is consistent with the improved healthy characteristics of milk from animals fed almost exclusively on pasture, compared to confined animals, as previously reported [53]. The significantly higher level of 18:1 in the LL cows was evident only in the extensive system, thus demonstrating a significant genotype x feeding interaction. The lower level of energy input in the extensive system, compared to the semi-intensive system, could somehow justify this result. In fact, Bitman et al. [54] found that C18:1 cis increased after a short term administration of bST when the cows were in negative energy balance; however, the differences with the untreated cows gradually decreased as energy balance became less negative.

## 5. Conclusions

In the present study, novel information about the GH p.L127V polymorphism and its interaction with the feeding system was investigated, for the first time in the Modicana breed. In particular, how different levels of pasture in the diet could interfere with the GH polymorphism on milk traits and fatty acid composition was studied. The tendency of LL cows towards higher levels of total unsaturated and monounsaturated fatty acids seems to suggest a possible role of the GH polymorphism on improving the healthy quality of milk. This research, conducted on a local breed reared in traditional systems, represents a first approach to the study of the role of the GH p.L127V polymorphism on the acidic composition of milk, and could represent a starting point for its extension to cosmopolitan breeds

## Figures and Tables

**Table 1:** Genotypes and alleles frequencies at the *GH* p.L127V in Modicana cows.

Growth Hormone *GH* p.L127V						
	N	LL	LV	VV	L	V
SI Farm	118	73	43	2	0.80	0.20
		(0.62)	(0.36)	(0.02)		
EX Farm	70	47	21	2	0.82	0.18
		(0.67)	(0.30)	(0.03)		
Total	188	120	64	4	0.81	0.19
		(0.64)	(0.34)	(0.02)		

SI, semi-intensive; EX, extensive.

**Table 2:** Effects of growth hormone (*GH*) p.L127V genotype and feeding system on milk yield and composition in the Modicana cow.

	*GH* Genotype (G)		Feeding System (S)		Significance			SEM
	LL	LV	EX	SI	G	S	G × S	
Milk yield kg/d	9.49	9.72	8.59	10.6	0.680	0.001	0.188	3.67
Fat %	4.09	3.90	4.00	3.99	0.250	0.962	0.856	0.287
Protein %	3.64	3.67	3.57	3.74	0.643	0.010	0.990	0.063
Lactose %	4.45	4.46	4.57	4.33	0.871	0.001	0.227	0.034
Casein %	2.78	2.78	2.75	2.81	0.952	0.338	0.560	0.046

EX = extensive system; SI = semi-intensive system. a, b, *p* < 0.05.

**Table 3:** Effects of *GH* p.L127V genotype and feeding system on milk fatty acid composition.

	*GH* Genotype (G)		Feeding System (S)		Significance			SEM
	LL	LV	EX	SI	G	S	G × S	
4:0	1.99	2.18	2.24	1.94	0.084	0.006	0.387	0.129
6:0	1.61	1.78	1.82	1.58	0.041	0.006	0.210	0.076
8:0	1.14	1.25	1.26	1.13	0.046	0.021	0.176	0.035
10:0	2.74	3.01	2.93	2.81	0.062	0.400	0.142	0.220
12:0	3.32	3.57	3.28	3.61	0.104	0.038	0.166	0.261
12:1	0.20	0.21	0.17	0.24	0.699	0.001	0.177	0.001
14:0	11.1	11.7	10.7	12.1	0.114	0.001	0.316	1.337
15:0 *iso*	0.40	0.40	0.36	0.45	0.891	0.001	0.195	0.003
15:0 *anteiso*	0.79	0.80	0.77	0.82	0.839	0.063	0.353	0.007
14:1 *c9*	0.90	0.90	0.68	1.12	0.918	0.001	0.915	0.070
15:0	1.58	1.59	1.58	1.59	0.830	0.661	0.081	0.014
16:0	29.2	28.9	26.8	31.3	0.708	0.001	0.861	7.784
17:0 *iso*	0.47	0.46	0.44	0.48	0.360	0.003	0.374	0.002
17:0 *anteiso*	0.59	0.56	0.46	0.70	0.350	0.001	0.164	0.008
16:1 *c9*	1.47	1.28	1.17	1.59	0.023	0.001	0.596	0.077
17:0	0.76	0.73	0.79	0.69	0.307	0.002	0.173	0.011
18:0	9.15	9.16	9.96	8.36	0.980	0.001	0.519	1.643
18:1 *t11*	1.42	1.45	2.12	0.75	0.757	0.001	0.594	0.096
18:1 *c9*	20.9	19.3	19.9	20.2	0.016	0.679	0.038	4.734
18:2 *c9c12*	1.65	1.64	1.63	1.66	0.819	0.583	0.095	0.041
20:0	0.22	0.20	0.20	0.22	0.223	0.137	0.541	0.002
18:3 *c9c12c15*	0.81	0.81	1.14	0.49	0.988	0.001	0.702	0.016
18:2 *c9t11*	0.69	0.66	0.93	0.42	0.584	0.001	0.908	0.018
SFA	60.7	61.9	59.4	63.2	0.259	0.001	0.551	0.570
MUFA	24.6	22.9	23.4	24.2	0.013	0.247	0.053	0.334
PUFA	3.61	3.70	4.37	2.94	0.491	<0.001	0.659	0.115
UFA	30.2	28.5	30.5	28.2	0.035	0.005	0.104	0.409
UFA/SFA	0.50	0.47	0.52	0.45	0.141	0.001	0.313	0.080
AI	2.76	3.08	2.73	3.10	0.061	0.033	0.149	0.086

EX = extensive system; SI = semi-intensive system. SFA, saturated fatty acids; MUFA, monounsaturated fatty acids; PUFA, polyunsaturated fatty acids; UFA, unsaturated fatty acids; AI, atherogenic index [23]. a, b, *p* < 0.05.
